# Supramaximal high-intensity interval training for older adults in a community setting: a pragmatic feasibility study

**DOI:** 10.1186/s11556-025-00379-6

**Published:** 2025-07-28

**Authors:** Erik Frykholm, Beatrice Pettersson, Mattias Hedlund, Maria Wiklund, Bengt Johansson, Carl-Johan Boraxbekk, Erik Rosendahl, Nina Lindelöf

**Affiliations:** 1 Department of Community Medicine and Rehabilitation, Umeå University, Umeå, Sweden; 2 Umeå School of Sport Sciences, Umeå University, Umeå, Sweden; 3 Department of Surgical and Perioperative Sciences, Umeå University, Umeå, Sweden; 4 Institute for Clinical Medicine, Faculty of Medical and Health Sciences, University of Copenhagen, Copenhagen, Denmark; 5 Institute of Sports Medicine Copenhagen (ISMC) and Department of Neurology, Bispebjerg Hospital, Copenhagen, Denmark; 6 Department of Diagnostics and Intervention, Diagnostic Radiology, and Umeå Center for Functional Brain Imaging (UFBI), Umeå University, Umeå, Sweden

**Keywords:** HIT/HIIT, Very vigorous exercise, Implementation, Aged, Ageing

## Abstract

**Background:**

Supramaximal high-intensity interval training (HIT) programmes can be challenging to replicate outside research settings. This study aimed to explore its feasibility for older adults in a community setting, incorporating perspectives from exercise participants and instructors.

**Methods:**

A pragmatic feasibility study using a convergent mixed- methods design involving four exercise instructors from one training facility and 21 older adult exercise participants (14 women, age-range 65–78). The previously used HIT programme consisted of 20-minute sessions that included a warm-up, ten 6-second intervals, and cool-down. Instructors first adapted the programme around these core components to their setting and then conducted 25 sessions. Both qualitative (individual interviews) and quantitative (estimated maximal oxygen consumption, estimated 6-second power, exercise- related motivation, and self-efficacy) data were collected and analysed (content analysis and descriptive statistics) in parallel. A taxonomy for implementation outcomes was used as an analytical matrix.

**Results:**

Experiences of both participants and instructors revealed that the structure of the training was regarded as engaging, enjoyable, and supportive for establishing routines and promoting ownership of training progression. Participants found personalised and motivating approaches to engage with the programme and confidence in their abilities grew. Changes in exercise-related motivation and self-efficacy showed individual variation without a group trend. Participants who completed the intervention showed a positive median change in estimated 6-second power and maximal oxygen consumption, although individual responses varied. Experienced challenges included coordinating tasks during intervals and confidence in managing the programme. Barriers to fidelity and to scale-up were related to the practical complexity and fixed structure.

**Conclusions:**

Supramaximal HIT can be implemented for older adults in a community setting with appropriate support, including individualised watt-based intensity and structured progression. The findings highlight how participants took ownership of their intensity progression, enabling them to challenge their limits. However, the fixed structure and complexity in managing short intervals may pose barriers to broader adoption. To enhance feasibility and scalability, simplifying interval management and providing clear, structured guidance are recommended. These insights help refine and optimize supramaximal HIT implementation for older adults in community settings.

**Trial registration:**

Open Science Framework 31 January 2023 (10.17605/OSF.IO/B7T2G).

**Supplementary Information:**

The online version contains supplementary material available at 10.1186/s11556-025-00379-6.

## Background

Regular physical activity and exercise have been shown to slow the decline in cardiovascular, metabolic, muscular, and cognitive functions associated with ageing [1–4]. Regular physical activity and exercise also prevent common diseases among older adults, such as cardiovascular disease, stroke, diabetes, and cancer [5]. Despite this, many older adults are inactive [5, 6], sometimes due to a perceived lack of time and sometimes due to beliefs about the irrelevance and inefficacy of physical activity and exercise [7]. The efficacy of physical activity and exercise is related to duration and intensity [8, 9]. Shorter sessions of high-intensity interval training have been shown to have similar to superior cardiovascular adaptations, compared to longer moderate-intensity training sessions [9–11], and have also been found to be enjoyable for both younger and older adults [12]. High-intensity interval training generally refers to intervals conducted close to the maximal heart rate or the maximum aerobic power, i.e., the power produced at peak oxygen consumption during a graded exercise test [13]. Previous research about high-intensity physical activity for older adults has mostly been focused on this specific type of high-intensity interval training [10]. However, intervals can also be even shorter and conducted at absolute intensities above maximal aerobic power, referred to in this study as supramaximal high-intensity interval training (supramaximal HIT) [14].

The benefits, acceptability, and appropriateness of supramaximal HIT are promising for older adults [15–18]. A specific programme with controlled, rather than all-out, supramaximal intensity has been developed for older adults [14] and later evaluated in a randomized controlled trial (RCT) with older adults previously not engaged in regular exercise [15, 19]. In the RCT, supramaximal HIT elicited a larger effect on leg strength and working memory compared to moderate-intensity training [15]. Additionally, supramaximal HIT elicited fewer adverse events and more positive events compared to moderate-intensity training [19]. There were no significant differences in the positive change in maximal oxygen consumption and blood pressure between groups, despite the supramaximal HIT group having only half the training time [15]. While this suggests that supramaximal HIT is both effective and time-efficient for older adults. The transition to community settings poses significant challenges [20, 21]. In such settings, factors such as participant adherence, variation in instructor experience, and logistical constraints may influence the effectiveness of an intervention [22]. Even interventions with strong support in controlled trials may face difficulties in practical implementation, leading to reduced effectiveness [23].

To address the transition, the assessment of implementation outcomes—defined as the measurable results of efforts to introduce and sustain new practices in routine settings—is essential [24]. These outcomes help differentiate between issues related to the intervention’s content and those arising from its execution. The success of an intervention in a real-world setting depends on several factors, including the fidelity of its delivery, the acceptability among participants, and the contextual fit of the programme. Feasibility studies are crucial in identifying potential barriers and facilitators to effective implementation and provide insights into the practical challenges of translating research findings into everyday practice [23]. The objective of this study was to explore the feasibility of supramaximal HIT for older adults in a community setting with perspectives from exercise participants and exercise instructors.

Specific research questions related to the feasibility of supramaximal HIT were:

1. What are the acceptability and appropriateness of the exercise program?
2. What are the participants’ attitudes and experiences from the program?
3. What are the fidelity and adherence to the program?
4. Are previously shown quantitative exercise effects maintained in the new context?

## Methods

### Study design

This was a single-arm pragmatic feasibility study, part of a larger project aimed at evaluating supramaximal HIT for older adults. A previously used programme [14, 15] was adapted in collaboration with exercise instructors. We used a convergent mixed-methods design [25] in which qualitative and quantitative data were collected and analysed in parallel [26] and thereafter integrated by the use of an analytical matrix (taxonomy) reflecting implementation outcomes [25]. The study was approved by the Swedish Ethical Review Authority (Ref. no.: 2022-06401-01), pre-registered at Open Science Framework [27], and the results are reported in accordance with the consolidated criteria for reporting qualitative research (COREQ) guideline [28]. In the pre-registration the sample size was determined to *n* = 26. The sample size calculation was based on a previous RCT showing a within-group change in maximal oxygen consumption of 1.4 (mL/kg/min), a standard deviation of 2.4 [15], 80% power and a 0.05 significance level. This sample size was not reached during the available time for recruitment and therefore only descriptive statistics were used.

### Participants and recruitment

Participants were both exercise participants and exercise instructors. Exercise participants were recruited with local advertisements through advocacy groups with interests in health for older adults and through the research group network. Eligible exercise participants were adults aged 65 years or older, at the time not involved in structured vigorous training, and with the ability to undertake a twice-weekly training programme for three months. Exclusion criteria were movement-related dysfunction, heart or lung conditions with exercise-induced symptoms, poorly controlled or untreated arterial hypertension, insulin-treated diabetes, or chronic and progressive neurological disease. Potential exercise participants provided written informed consent and underwent a medical examination led by an experienced cardiologist (BJ), including 12-lead electrocardiography and blood pressure, before inclusion.

Four exercise instructors were purposefully recruited through management using convenience sampling from the staff with permanent positions at a local training facility with 20,000 members and an extensive selection of group sessions. The selected instructors, comprising three women and one man, 37 to 53 years of age, and with 12 to 18 years of varied experience in group exercise instruction.

**Fig. 1:**
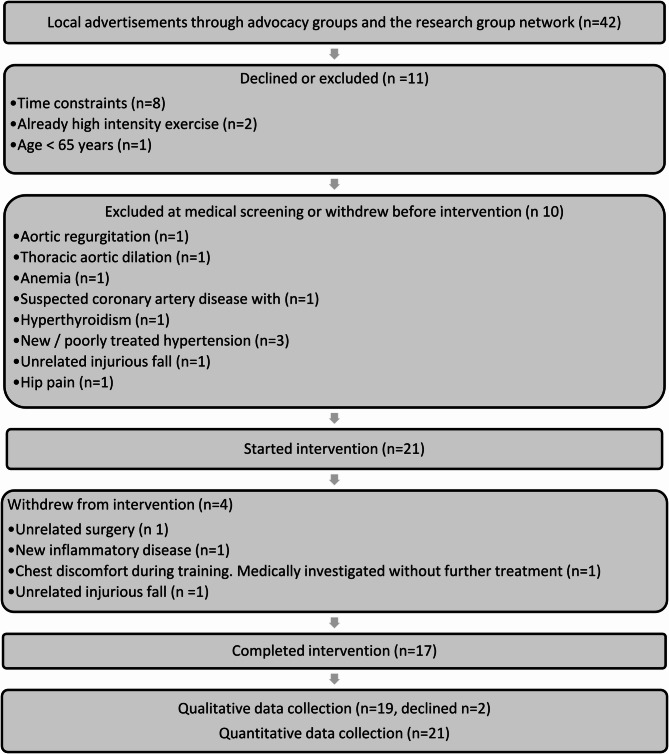
Exercise participant flow

Figure 1 describes participant flow in the study and Table 1 describes participants at baseline. Twenty-one exercise participants (14 women and seven men) started the intervention and were divided into two exercise groups based on preferred time. Nineteen of 21 participants self-reported some exercise experience in adult age. Examples from an open-ended follow-up question were mixed and ranged in intensity from walking to various group training formats at training facilities and in recreational sport activities.

**Table 1:** Characteristics of exercise participants at baseline

	*n*	median	min	max
Age (years)	21	70	65	78
Education (years)	19	16	10	23
Height (cm)	21	165	150	186
Weight (kg)	21	82.6	60.1	96.4
BMI (kg/m^2^)	21	28.7	23.4	39.6
Blood pressure (mm Hg)				
Systolic	21	135	100	140
Diastolic	21	85	68	90
Medical diagnoses (n)	21	2	0	5
Medications (n)	21	3	0	9
Estimated maximal 6-second power (Watt)	21	376	196	560
Physical activity minutes (min/week)	21	210	75	390
Estimated maximal oxygen consumption (ml/kg/min)	21	26	19	35
S-ESES (10–40 points) ^a^	20	27.5	17	36
BREQ-2, RAI (−20 to 24 points) ^b^	19	9.0	−5.2	20.0

^a^ Higher scores indicate a stronger belief in one’s ability to exercise and be physically active

^b^ Higher scores indicate greater relative autonomy

*BMI* Body Mass Index, *BREQ-2* Behavioral Regulations in Exercise Questionnaire-2, *S-ESES* Swedish Exercise Self-Efficacy Scale

### Exercise intervention

The original supramaximal HIT programme was repeated twice weekly for a total of 25 sessions for three months. One session took 20 min and consisted of warm-up, cool-down, and ten 6-second intervals on indoor bicycles with 54 s of rest between intervals. The programme is described in detail in Simonsson et al. [15].

Core components kept from the original protocol [15] included the 6-second intervals, that exercise intensity was individually prescribed as external target power output in absolute watts, and was regulated by standardized pedal frequency and individual adjustment of the brake resistance. The interval target power output started at 60% of the participant’s maximum mean 6-second power output, estimated at baseline with a modified Borg Cycle Strength Test [29]. The intensity was the same for all the ten intervals within-session but was allowed to change, up or down, after each session by pre-defined criteria. To increase resistance, the participant should have (1) the ability to maintain standardized pedal cadence during the session and judge that one additional interval would have been possible to perform; (2) a rating of perceived exertion (RPE) less than ‘very hard’ [30]; and (3) a wish to increase intensity.

A notable pre-decided deviation from the original protocol [15] was the removal of support from two supervisors during training. In the previous study, the supervisors prepared the equipment, monitored the training, and collected data related to adherence and interval intensity, as well as change criteria. Between sessions, they adjusted interval intensity accordingly. In this study, the tasks were managed by the exercise participants themselves receiving only standard guidance typically provided by instructors during regular sessions at the training facility.

### Up-skilling and co-design of the exercise programme

Before the training intervention period, the exercise instructors from the training facility took part in one up-skilling session and one co-designing workshop. Up-skilling involved learning the core components of the supramaximal HIT programme [15]. After up-skilling, they were asked to participate in a co-designing workshop to further adapt the programme. The intention was to make the programme as applicable as possible within the context of the specific training facility.

The co-designing process involved developing the logbooks used, session planning and verbal instructions that could be personalized during exercise sessions, suitable music using the specific equipment, and regulations applicable to the setting. They also reduced the cool-down time (2 min) in favour of a longer warm-up (8 min) including brief increases in cadence and resistance, respectively (as opposed to the previously used constant load for 5 min). This was done for consistency with the interval nature of the rest of the session. The decision was made to keep a previously used visual timer screen but remove the sound to be able to provide more verbal instructions. The exercise instructors rotated to allocate 45 min of working time to each 20-minute session, with two sessions (for the two groups of participants) conducted back-to-back on Wednesday and Friday mornings.

### Data collection

**Fig. 2:**
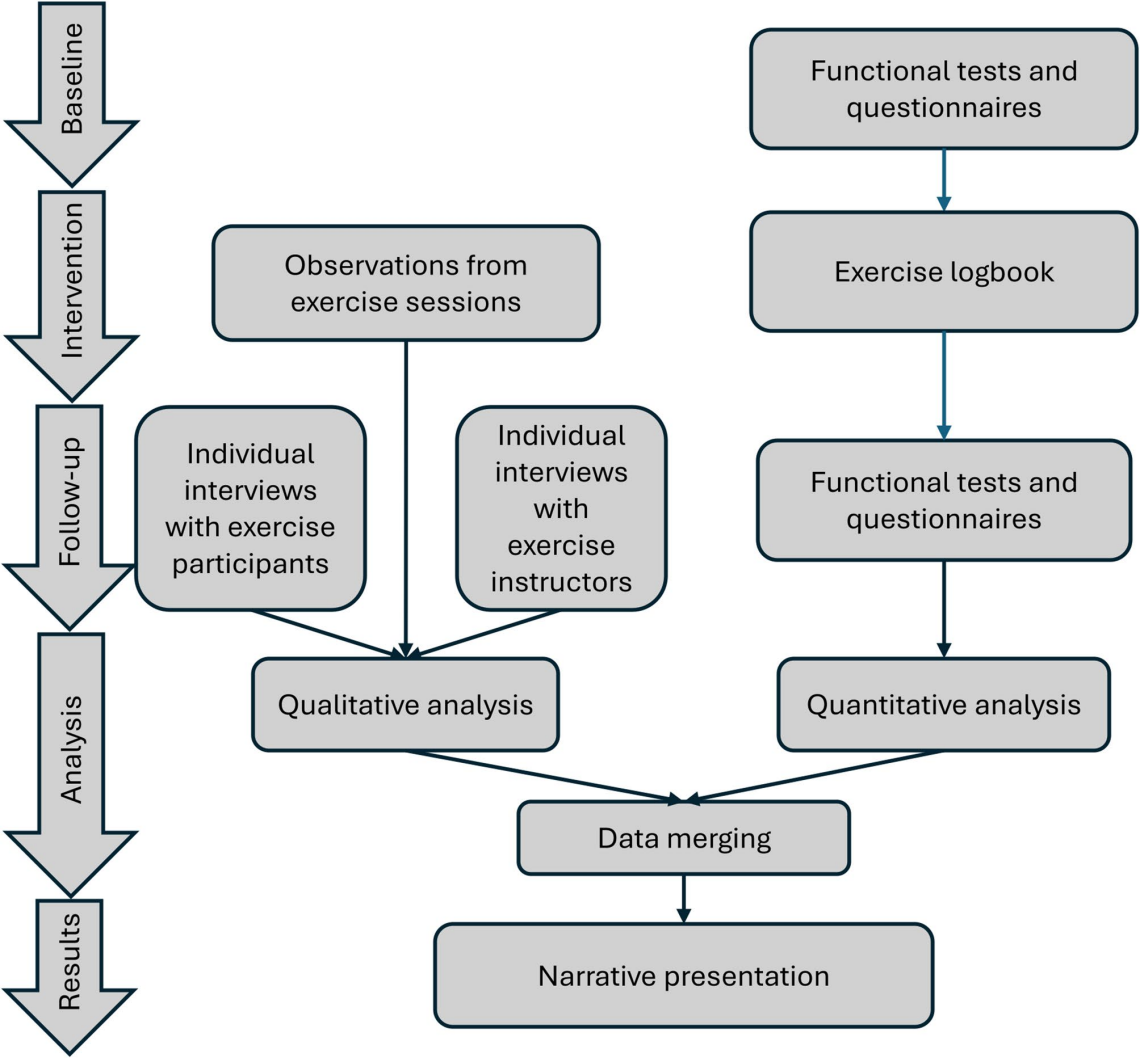
Overview of the mixed-methods process, from data collection at baseline and follow-up, the intervention period (25 sessions over 3 months), and parallel analyses to integration of findings through a narrative weaving approach

Multiple methods were used for data collection. Interviews were used to address all research questions, complemented by questionnaires, physical tests, and observations. Thereafter were all data integrated in the analytical matrix (taxonomy) reflecting implementation outcomes. At the start of the study, descriptive data of participant characteristics were collected from both exercise participants and instructors through a questionnaire. Exercise participants also completed questionnaires assessing their exercise self-efficacy, exercise motivation, and physical activity, and conducted two physical tests. During the intervention period, researchers (*BP and EF*) conducted observations on two training sessions. Following the intervention, individual interviews were conducted (Fig. 2).

#### Qualitative data collection

All exercise participants who had started the intervention (*n* = 21) and all exercise instructors were invited to participate in an individual interview after the intervention. Nineteen exercise participants and all four exercise instructors accepted. The reasons for declining participation in the interview stemmed from a sense of not having engaged enough (*n* = 1) and ongoing medical evaluation (*n* = 1).

All the interviews were conducted by BP, who has expertise in qualitative interviewing. She had no relationship with the participants prior to the study and they were only informed that she was employed within the study. All interviews with the exercise participants were conducted in a lab environment designed as an apartment, which facilitated a relaxed interview environment. Three of the interviews with the exercise instructors were held in a conference room at their workplace, and one via video conference call. The interviews were conducted between May and June 2023. Interviews with the exercise participants lasted 20–80 min (median 46 min) and 34–49 min (median 45 min) with the exercise instructors. The interviews were all audio-recorded and transcribed verbatim by a professional transcriber.

Semi-structured interview guides with open-ended questions were used, one for the exercise participants and one for the exercise instructors. Both interview guides were informed by the taxonomy of implementation outcomes [24] and included questions about how the intervention was accepted and fitted into the context of a group training programme at the local training facility. In addition, they explored barriers and facilitators at both individual and organizational levels (Supplementary file 1). All interviews also explored the participants’ experiences of and attitudes toward the intervention.

Observations were conducted during sessions 17 and 18. On the first occasion, the focus was on group dynamics, including the role of exercise instructors, their support for participants, and participant interactions, as well as routines surrounding the exercise. The second occasion focused on whether participants followed instructions regarding cadence, resistance, and effort levels. Field notes from both observations were included in the qualitative analysis.

#### Quantitative data collection

All quantitative pre- and post-data collection was done during one visit at each timepoint conducted by EF. The data collection was performed at an exercise lab (Umeå Movement and Exercise Laboratory), during two weeks before (pre) and after (post) the intervention. The visit lasted about one hour and was scheduled during daytime based on participant preferences. The timeline of the data collection followed the description below.

Maximal oxygen consumption was estimated using Ekblom-Bak tests conducted on a Monark cycle ergometer (Monark 839E, Monark, Vansbro, Sweden) with continuous heart rate monitoring (Polar H10 heart rate sensor, Polar, Kempele, Finland). Total duration of the test was 8 min, with an initial 4-min stage at a fixed work rate, directly followed by a higher individualized work rate for 4 min [31]. Maximal oxygen consumption was estimated using published equations that have been found to explain 86 and 83% of the variation (adjusted R-squared) in maximal oxygen consumption in men and women respectively with a coefficient of variation of 8.7% and standard error of the estimate of 0.28 L/min [31].

Physical activity was assessed with the Swedish National Board of Health and Welfare indicator questions regarding physical activity with categories and translated to activity minutes for interpretability [32]. The indicator questions have been shown to detect insufficient physical activity accessed with accelerometers with 63% sensitivity and 67% specificity in adults [32].

Exercise-related self-efficacy was assessed using the Swedish Exercise Self-Efficacy Scale (S-ESES) [33, 34]. The S-ESES contains 10 items with a four-point scale ranging from 1 (not at all true) to 4 (always true). The scores are summed together, ranging between 10 and 40, with higher scores indicating a stronger confidence in one’s ability to exercise. The S-ESES has in a Swedish sample shown very high-reliability with an intraclass correlation coefficient of 0.92 and standard error of measurement of 5.3 points [33].

Exercise-related motivation was assessed using the Swedish version of Behavioral Regulations in Exercise Questionnaire-2 (BREQ-2) [35–37]. This questionnaire consists of 19 items with a five-point Likert scale ranging from 0 (not true for me) to 4 (very true for me). First, a unit-weighted composite score was computed for each of the five factors. Second, a relative autonomy index (RAI) was calculated with the score from each factor weighted and summed, ranging from − 24 to 20, with higher scores indicating greater relative autonomy [36, 38]. An increase in relative autonomy has been shown to be indicative of future engagement in physical exercise [39].

Expectation of training adaptations was assessed with a single question, ‘Do you expect this form of training to improve your aerobic fitness?’, with a five-point scale ranging from 1 (No) to 5 (Yes, very much).

Maximal 6-second mean power output was estimated with a modified Borg Cycle Strength Test [29] using Tomahawk IC7 indoor bicycles (Indoor Cycling Group, Nürnberg, Germany). In our adapted version of the test, 30-second incremental work stages were intercepted with 30 s of active rest and continued until failure to complete a work stage or until a rating of ‘very hard’ on the Borg RPE scale [14, 30]. Partially completed stages were added to the highest completed stage proportionally to give a continuous result in watts [40]. The maximum 6-second mean power output was estimated as 1.75 times the 30-second performance [14]. In the original version the estimated maximal 30-second power had a correlation (Pearson’s r) of 0.90 to maximal performance [29].

After each session were exercise participants encouraged to note attendance, external target power output in absolute watts, use of escalation guidelines, ratings of perceived exertion [30], and any adverse event in a logbook and separate adverse event forms. The exercise participants could also select if the adverse event was related to by any or several of the following: general vitality, equipment, disease, musculoskeletal condition, cardiorespiratory condition, or emotional reactions related to the session itself. Adverse events were defined as serious if resulting in death, risk of death, hospitalization, disability or permanent damage, required an intervention to prevent disability or permanent damage, or other serious medical events. The logbook was analysed after the training period regarding target power output across sessions, peak RPE, and adverse events.

### Analysis

We used a taxonomy for implementation outcomes to deductively analyse and integrate qualitative and quantitative results, aiding the translation of our findings into terms relevant for future implementation. The taxonomy was developed by Proctor et al. [24] through synthesis of the literature and expert consensus. The taxonomy includes eight categories: acceptability, adoption, appropriateness, cost, feasibility, fidelity, penetration, and sustainability. It can be applied regardless of the underlying implementation theory, covering a wide range of outcomes [24]. Although participant perspectives are traditionally not included across all categories, we recognized the unique insights gained by incorporating both participant and instructor perspectives into each outcome. This approach enhances the relevance of our findings for practical implementation, particularly in promoting self-management within exercise interventions.

In line with the convergent mixed-methods design, qualitative and quantitative data were analysed in parallel after the intervention (Fig. 2) [26], and integrated through a merging approach [25]. This involved combining findings from both methods for overlapping concepts, such as presenting verbal expressions of exercise motivation alongside self-reported questionnaire data on the same topic [25]. Proctor et al.’s taxonomy of implementation outcomes [24] guided both the deductive qualitative analysis and its integration with the quantitative data. A qualitative inductive analysis generated subcategories, into which the relevant quantitative data were incorporated during the narrative phase of result presentation.

*Qualitative analysis* of the interviews and field notes employed a two-stage qualitative content analysis methodology: initially deductive guided by the taxonomy of implementation outcomes [24], followed by an inductive approach. Qualitative content analysis helped examine details in the data by highlighting differences and similarities within the codes and categories [41]. The deductive approach was used to explore pre-existing knowledge in a new setting [42], while the inductive method, guided by the data, helped us discover new insights by identifying recurring patterns and moving towards a deeper understanding within each construct of the used taxonomy [43].

Before the deductive phase, a coding sheet was established by the authors based on the taxonomy of implementation outcomes [24] (Supplementary file 2). The taxonomy includes eight categories, but the categories of cost and penetration were excluded from the coding sheet as these elements were not covered by the intervention. BP coded the material and concurrently sorted the codes according to the coding sheet.

After completing the deductive phase, an inductive stage was commenced to compare and contrast the codes, leading to the formation of subcategories within each category concerning the taxonomy of implementation outcomes. Data analysis was conducted using MAXQDA 2020 software. Throughout the analysis process, triangulation through ongoing discussions among four authors (BP, EF, MW, and NL) ensured consensus regarding both the coding sheet and the results of the analysis. Additionally, to enhance credibility, the four authors independently performed analyses on one interview each, subsequently discussing their interpretations collectively.

*Quantitative analyses* were done descriptively with R [44] and RStudio [45], using tidyverse packages [46].

## Results

The results, primarily guided by the qualitative data, are presented according to the taxonomy of implementation outcomes [24]. As a result of the deductive analysis, we identified expressions corresponding to all implementation outcomes outlined in the coding sheet. These serve as categories in the presentation of the results. The qualitative inductive analysis generated 16 subcategories within these categories (Table 2). Direct quotes in the text are indicated as ‘EP’ for exercise participants, followed by gender along with their respective interview number. Quotes from exercise instructors are denoted as ‘EI,’ without specifying gender, due to the small number of instructors.

**Table 2:** Overview of the data collection methods and contribution to the results in the convergent mixed-methods design

Implementation outcomes	Qualitative data		Quantitative data
	Categories	Subcategories	
Adoption	Grounds for training engagement	Expectations from engagement	Expectation of training adaptation on aerobic capacity
		Need for support	
Appropriateness	Continuous appraisal of suitability	Approval of concept	
		Beliefs in own capabilities	
Acceptability	Motivational synergy of acceptance	Translation of experience	Exercise-related self-efficacy
		Value through assessment	Maximal oxygen consumption
		Create personal relevance	Exercise-related motivation Maximal 6-second power
Feasibility	Agency in training	Learning curve	Training logbook
		Value of clear guidance	
		Responsibility of exercise management	
		Shared experiences	
Fidelity	Pursuit of accuracy	Commitment to training	Training logbook
		Personalized strategies for accuracy	
		Implement meaningful modifications	
Sustainability	Continuity of supramaximal HIT	Considerations of continuation	
		Reflections on suitability	

### Adoption– grounds for training engagement

The decision to adopt and explore supramaximal HIT among exercise participants was driven by expectations of enjoyment, health benefits, and contributing to research. A desire to engage in exercise was conveyed along with a perceived need for structure and support.

The exercise participants described diverse ‘expectations from engagement’ in supramaximal HIT and shared various motives. Some sought novelty and enjoyment outside their comfort zone, while others were driven by the prospect of health improvement and appreciated the study’s initial health examination. When asked about expected improvements in aerobic fitness, responses varied from ‘Yes, a little’ to ‘Yes, very much’, with ‘Yes, much’ as the median response. Contributing to research on older adults’ health was also highlighted.

*‘If something positive comes out of this*,* that my part in it all could potentially contribute to the future well-being of older adults. Well*,* then that’s excellent. I think that would be absolutely fantastic.’* (EP1, Woman).

Exercise participants expressed a ‘need for support’ in both initiating and maintaining exercise, which they acknowledged as necessary. This need often stemmed from a lack of motivation.

*‘The kids gave me a treadmill. (Sighs) I’ve had two exercise bikes in my life… I was supposed to exercise*,* but of course then I didn’t. Motivation for training is so damn hard.’* (EP8, Woman).

Unfamiliarity with gym environments and group sessions was highlighted as an additional barrier for adoption. The supramaximal HIT programme was hoped to provide essential motivational and competence support, with scheduled sessions expected to maintain personal commitment and group accountability.

### Appropriateness– continuous appraisal of suitability

The appropriateness of supramaximal HIT for older adults was continuously assessed throughout the study. Opinions varied, influenced by personal beliefs, physical capabilities, and health status.

The exercise participants expressed ‘approval of the concept’ of supramaximal HIT for its theoretical simplicity, effectiveness, and time efficiency, appreciating the structured and professional environment. Opinions on the practical suitability varied. Some older adults saw it as universally beneficial, while others felt it suited those with prior exercise experience. Challenges during training, fatigue, and negative beliefs also emerged.

‘*It’s quite challenging…one must train intensely for a short period. For people my age and older*,* not everyone may be able to cope with it’* (EP7, Man).

These challenges were mitigated through trust in the concept and peer support. The exercise instructors generally agreed that supramaximal HIT may suit many older individuals, emphasizing that it is not reserved for younger people: ‘*older adults are not made of glass*’ (EI4).

Suitability was often linked to the exercise participants’ ‘beliefs in their own capabilities’, shaped by previous experiences. This could be dependent on health status, as diseases or pain set limitations for physical activity and exercise. Therefore, some were pleasantly surprised by their ability to train with such a high intensity, enhancing the perceived appropriateness of supramaximal HIT.

*‘It was really difficult. But*,* I mean*,* I thoroughly enjoyed being a part of it anyway. (laughs) I have a hard time with my rheumatoid arthritis*,* I can’t go for walks. There’s a lot with my feet*,* so cycling and being in the water are good for me […] and it only lasted 20 minutes.’* (EP9, Woman).

### Acceptability – motivational synergy of acceptance

The acceptability of supramaximal HIT among exercise participants was interpreted as driven by a motivational synergy, where personal exercise experiences, perceived outcomes, and personal relevance converged. This combination fostered perceptions of supramaximal HIT as an agreeable and satisfactory form of exercise.

*‘*Translation of experience’ was reflected by the varied experience of group exercise or more strenuous physical activity among the exercise participants. Those with more exercise experience felt comfortable with the high intensity and saw it as an advantage, knowing their physical limits and enjoying the challenge. Exercise instructors also noted that experienced exercise participants found it easier to establish a rhythm in their sessions and push their limits. In contrast, coaching exercise participants without prior vigorous training experience was perceived as more challenging. Self-reported exercise-related self-efficacy suggest no group change but considerable individual variation in S-ESES (median [min; max], 0 [−13; 5], Table 3).

**Table 3:** Absolute and relative changes in quantitative outcomes

Change in	*n*	median	min	max
Estimated 6-second power (Watt)	17	42.0	−10.5	144.4
Estimated 6-second power (%)	17	13.0	−3.0	60.0
Estimated maximal oxygen consumption (ml/kg/min)	17	0.6	−2.3	4.3
Estimated maximal oxygen consumption (%)	17	2.5	−9.3	16.8
S-ESES (10 to 40 points) ^a^	16	0.0	−13	5
BREQ-2, RAI (−20 to 24 points) ^b^	14	1	−4.0	4.2

^a^ Higher scores indicate a stronger belief in one’s ability to exercise and be physically active^b^ Higher scores indicate greater relative autonomy

*BREQ-2* Behavioral Regulations in Exercise Questionnaire-2, *S-ESES* Swedish Exercise Self-Efficacy Scale

*‘Guiding older people to dare to push themselves is challenging*,* especially if they don’t have much experience with it. To dare to reach that point where it’s actually quite tough. Of course*,* it’s up to each individual. But … you still see those who engage in small talk.’* (EI1).

The exercise participants described that the acceptability was partly influenced by ‘value through assessments’ and its outcomes. The format made them assess training adaptations during and after the intervention, which motivated them to continue training. Adaptations during sessions included increased or sustained resistance, or even perspiration.

‘*I wrote [in exercise diary]: sweat is now on my glasses. Because at first*,* you feel dampness under your fringe*,* and then it drips onto your glasses*,* and then it drips onto your shirt.’* (EP8, Woman).

Observing progress, such as increased resistance, indicated improvement but could also lead to disappointment if intervals could not be completed.

*For some reason*,* I just didn’t make it [own set goal of progression]. And honestly*,* I’m a little disappointed in myself for that. But*,* you know… since I had to keep up the intensity for all the intervals*,* I realized—I’m not going to get where I expected.’* (EP12, Man).

**Fig. 3:**
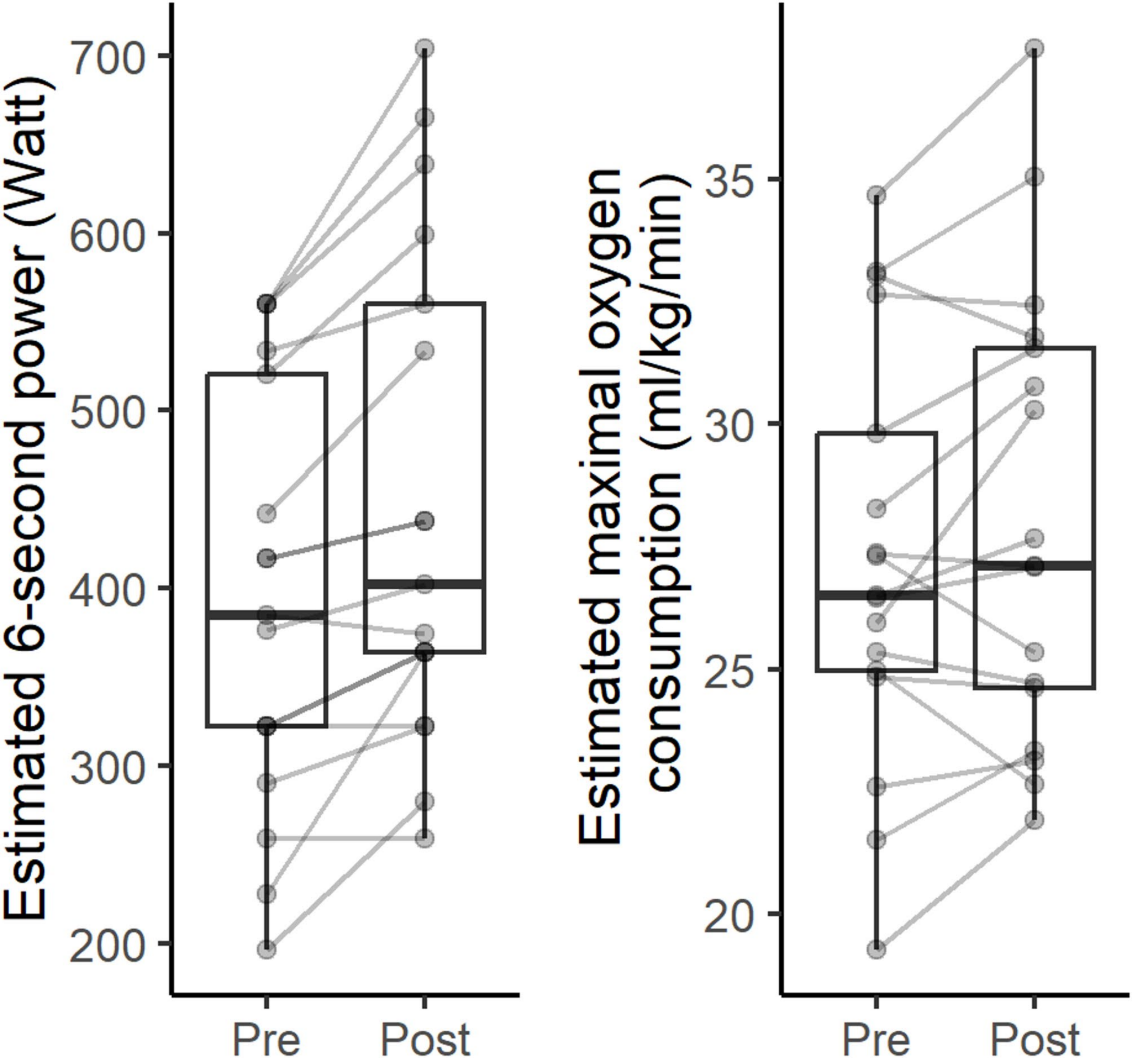
Estimated 6-second power (left) and maximal oxygen consumption (right) at pre- and post-intervention assessments. Boxplots visualizing the median, two hinges and two whiskers. The lower and upper hinges correspond to the first and third quartiles. The whiskers extend from the hinges to the largest and smallest value no further than 1.5 of the distance between the first and third quartiles from the hinges. All data are also plotted individually with baseline and follow-up values connected with thin lines

Other expressions of improvements were better overall aerobic capacity, more energy, sleeping better, or better endurance. Variation in improvement was also seen in quantitative outcomes such as change in estimated 6-second power and estimated maximal oxygen consumption (Table 3; Fig. 3).

A few participants expressed having unpleasant experiences related to the training, such as muscle soreness, or soreness from the saddle. Additionally, one adverse event was overheard during the observation and was encouraged by one of the researchers to report using the adverse event report form. Four of the 21 exercise participants reported an adverse event. In total, thirteen events were reported, none of which was classified as a serious adverse event. One or more selected categories were musculoskeletal (*n* = 8), equipment (*n* = 5), cardiorespiratory (*n* = 3), and disease (*n* = 1). Examples included hand, shoulder and saddle discomfort related to the specific equipment, chest discomfort (medically investigated without further treatment), and that the training increased symptoms (from inflammatory disease).

**Fig. 4:**
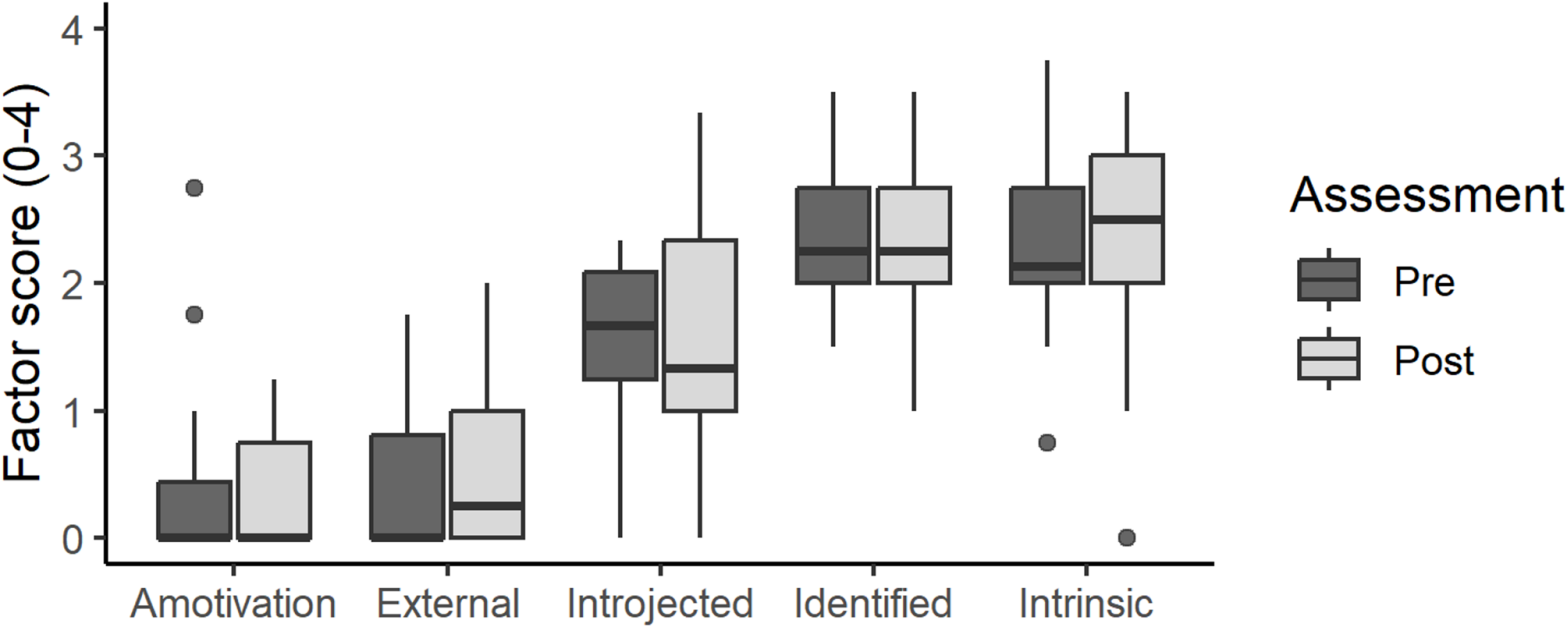
Behavioral Regulation in Exercise Questionnaire-2 score profile for each of the five motivational factors underlying the weighted and summated score. Measured at baseline and post-intervention assessments. Boxplots visualizing the median, two hinges and two whiskers, and all outlying points individually. The lower and upper hinges correspond to the first and third quartiles. The whiskers extend from the hinges to the largest and smallest value no further than 1.5 of the distance between the first and third quartiles from the hinges. Data beyond the ends of the whiskers are plotted individually

The exercise participants’ ability to ‘create personal relevance’ contributed to their motivation for the training. Initial motivations often persisted, and training was generally found to be fun and enjoyable. Completing sessions contributed to a sense of satisfaction and accomplishment. Participants’ motivational profiles, measured by the BREQ-2, indicated a predominantly autonomous motivation at both at baseline and the post-intervention assessments (Fig. 4). Changes in RAI scores after the intervention varied between − 4.0 and 4.2 with 1 being the median change (Table 3).

### Feasibility– agency in training

The feasibility of supramaximal HIT was assessed by participants’ perceived ability to self-manage the programme, learning individual and programme limits. Exercise participants discovered their preferences for guidance and felt supported by instructors and peers in taking ownership of their training.

Both the exercise participants and instructors described self-management as involving a ‘learning curve’. Initially, exercise participants required time to familiarize themselves with the bike and settings, and with exercise instructors providing practical assistance. As training progressed, interval management became easier and sessions more comfortable.

*‘They [the instructors] count down the seconds until it’s time to increase the pace and add resistance.It took us a while to get the hang of it. The intervals are so short; you have to turn the dial and concentrate to keep the pace up before those six seconds. It was a bit tricky. Even the instructors found it challenging at first*,* but now it works better. You learn as you go.’* (EP3, Man).

Some participants found self-management challenging throughout the training period and hesitated to seek help due to shame.

*‘I didn’t ask so many times because all the other participants seemed to be able to understand’.* (EP8, Woman)

The exercise participants highlighted the ‘value of clear guidance’ for completing the intervals and exploring their own and the programme’s limits. They appreciated diverse training guidance, such as the timer screen for an overview, instructor motivation for intensity, and bike displays for accuracy. Instructors provided educational guidance, and valuable support with interval management. Exercise instructors highlighted their commitment to providing clear guidance, conferring early in the training period to ensure consistency. Despite these efforts, they observed participants struggling to understand instructions and targeted tips. This was confirmed by some of the exercise participants, who emphasized their need for clearer guidance to fully engage with the programme.

Uncertainty about the focus of supramaximal HIT prompted questions about programme adjustments, leaving some exercise participants feeling unsupported in self-management when exercise instructors were unable to provide answers.

‘*I thought they should have been more explicit. They mentioned that it is not crucial to have 85 [in cadence]. In my opinion*,* either it’s important or it’s not (laughs)*,* you know?*’ (EP4, Man).

Another view was that:

‘*No one can misunderstand this; it has been clear what it is all about. That has been really helpful*.’ (EP18, Woman).

Many exercise participants embraced ‘responsibility for exercise management’, the key lying in understanding and learning their personal limits. Self-managing resistance progression was a notable task and could lead to frustration and shame when progress did not meet expectations.

*‘It’s difficult with this aspect of daily condition, knowing how you actually function when you’re really pushing yourself. You almost don’t know from time to time until you have started.’* (EP3, Man)

Participants constructed knowledge about their physical responses to training over time, supporting informed decisions about when to progress or lower intensity.

*‘I think someone else would be setting the bar too low. Because now I could push myself. I felt when… well*,* this is on the verge of my limits.’* (EP25, Man).

**Fig. 5:**
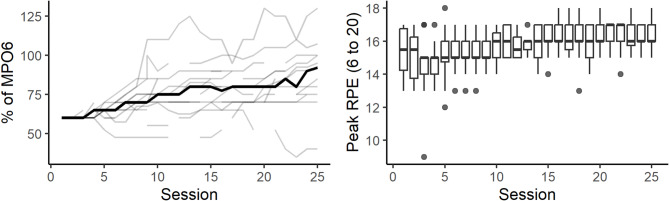
Training target power output across sessions relative to estimated 6-second power (% of MPO6, left) with individuals as thin lines and the group median as a thick line, and the peak RPE (right). RPE = Rating of perceived exertion. Boxplots visualizing the median, two hinges and two whiskers, and all outlying points individually. The lower and upper hinges correspond to the first and third quartiles. The whiskers extend from the hinges to the largest and smallest value no further than 1.5 the distance between the first and third quartiles from the hinges. Data beyond the end of the whiskers are plotted individually

Logbooks confirmed that participants managed their training intensity, changing the stipulated external load and noting peak ratings of perceived exertion over time (Fig. 5).

The exercise participants felt connected to each other through ‘shared experiences’, discussing their progress, challenges, and strategies both verbally and non-verbally. They sought advice on self-management and sometimes engaged in secret competition for motivation. Participants noted that both exercise instructors and the group dynamics influenced their motivation, performance, and enjoyment. While some felt a sense of belonging, support, and joy, others experienced negative feelings of competition and comparison. Exercise instructors confirmed strong group cohesion and the development of social bonds over time, reaffirming their prior beliefs about the importance of regularly meeting the same group.

*‘Well*,* in a way*,* I felt this was like a kamikaze mission because I’ve been to cycling training once and I almost passed out…But this was calm and controlled. And then there was a very nice group. We laughed a lot*,* and it wasn’t deadly serious. I thought we encouraged each other in a good way… so there was no kamikaze mission. (laughs)* (EP16, Woman).

### Fidelity– pursuit of accuracy

Fidelity was strived for through attendance monitoring and the implementation of strategies to ensure session fidelity and meaningfulness, reflecting a dedication to maintaining programme accuracy for both training benefits and research purposes. Exercise participants expressed a ‘commitment to training’, attending as many training sessions as possible. Those who completed the programme had a median (min; max) attendance rate of 88% (40%; 100%). Absence was usually due to pre-planned activities such as trips or medical appointments. Adherence reflected a commitment both to themselves and to the study, feeling a missed potential for improvement when sessions were missed.

*‘I actually have to attend the training*,* otherwise I won’t become any better.’ (EP4*,* Man)*.

When facing difficulties adhering to the programme, the exercise participants expressed devising ‘personalized strategies for accuracy’ based on preferences, feelings, and performance, or through group consultation. They often struggled with adjusting resistance promptly before the 6-second interval, affecting their ability to exert high effort. One strategy was to extend intervals or add extra ones.

*‘*You could add a second if you felt that it was taking too long to regulate the resistance*’* (EP9, Woman).

Adjusting resistance became more intuitive over time. Exercise participants learned to adjust resistance approximately or accept wider interval ranges rather than exact values. This was confirmed by the research team during observations. Another strategy was to increase cadence before interval onset to match the goal cadence when resistance was added. These strategies were informed by instructor guidance and experimentation.

*‘Maintaining [a cadence of] 85 while adjusting the control was challenging. So*,* instead*,* I started going up to 100*,* and by the time I fiddled with the control*,* I would be back at 85.’* (EP23, Woman).

Some exercise participants ‘implemented meaningful modifications’ and set their own training goals based on information provided by the bike’s display and prioritized reaching them over adhering strictly to the structure of the programme. This was evident during observations, where exercise participants could pedal at a constant cadence and only change resistance or vice versa, both increasing intensity between intervals. Instructors confirmed these observations and believed the modifications stemmed from a lack of understanding of the intervals. Other modifications were extending sessions by arriving early for extra pedalling or adding gym time before or after cycling.

*‘There were some guys we always met in the changing room*,* and we’d say*,* “Yeah*,* we really worked hard today!” (laughs) It has become a motivational thing*,* pushing ourselves to achieve a certain distance.’ (EP3*,* Man)*.

### Sustainability– continuity of supramaximal HIT

Sustainability highlights the transition from temporary to permanent inclusion of supramaximal HIT at the training facility, considering both exercise participants’ preferences and exercise instructors’ views on suitability.

Exercise participants had varying ‘considerations of continuing’ with supramaximal HIT. While some were motivated to continue with high-intensity training, maintaining supramaximal HIT independently posed challenges, which included lack of group motivation, membership fees, and adapting the training. Suggestions to facilitate continuation included enhancing support for self-management in different ways, through either fewer bike manoeuvres, exploring longer intervals, such as 10 s to easier achieve at least 6 s of high intensity, a digital application guiding the training, or programmable bikes. Conversely, despite an initial interest, some found themselves preferring outdoor activities and/or self-training.

*‘What would be really great is if you could somehow transfer this to a regular exercise bike*,* you know*,* like the ones people have at home but rarely use after a while. If there was a simple programme where you could track your progress*,* maybe through an app on your phone or something*,* I think people would be motivated to do it since it doesn’t take much time. Of course*,* it depends on the type of bike you have*,* so it might not be exactly the same*,* but something that turns this into a self-managed workout routine.’* (EP18, Woman).

The exercise instructors shared ‘reflections on the suitability’ of supramaximal HIT and how it could fit within the organization. They discussed the benefits of possibly forming permanent groups with a personal trainer over a period of time. While supramaximal HIT was valued for its short duration of hard work, they agreed that it most likely would be better with intervals that may be up to 30 s and/or fewer parameters to define intensity, and the overall fixed structure was seen as limiting its integration.

‘*If it’s 6-second intervals*,* perhaps you don’t need all these parameters at the same time*,* but instead just focus on feeling [of exertion]’* (EI2).

Proposals also included extended warm-up and cool-down periods. The exercise instructors believed that the suggested changes would enhance the value of the sessions for older adults and also make the training format more suitable for their organization.

## Discussion

This pragmatic study used a convergent mixed-methods design to assess the feasibility of a supramaximal HIT programme in a community setting, with a taxonomy on implementation outcomes as an analytical matrix. The experiences of both exercise participants and exercise instructors reveal that the structure of the training was regarded as interesting, enjoyable, and supportive for initiating and sustaining routines and promoting a sense of ownership over their training progression, which was also observed in the exercise participants’ logbooks. As confidence in their abilities grew, some found personalized and motivating approaches to engage with the programme, with individual variation in change on measures of exercise-related motivation or self-efficacy without a clear group trend. Some challenges arose, particularly in coordinating tasks during intervals, and differences in how confident participants felt in managing the programme. The majority of exercise participants that completed the intervention had some improvement in estimated 6-second power and estimated maximal oxygen consumption indicated by a positive median result. Lack of fidelity to the training programme and indicated barriers to scale-up were related to practical complexity and rigidity of the training protocol.

Acceptability of the programme was partly based on anticipated benefits, and the majority of exercise participants who completed the intervention also had an increase in estimated 6-second power and estimated maximal oxygen consumption. This was indicated by a positive median result, although individual responses varied. Previous studies that have implemented HIT in real-life settings [47] have focused on middle-aged and/or inactive adults [48–50]. Of these studies, one reported promising improvements in predicted maximal oxygen consumption [48], while another found only modest improvements [49], likely due to lower adherence [49]. In the present study, the adherence to the exercise programme resembled a previous RCT [15, 19], evaluating the HIT programme the present study was based on. However, the median improvement in estimated maximal oxygen consumption was about half that of the previous trial [15]. This despite similar improvements in estimated 6-second power [51]. The seemingly lower improvements in estimated maximal oxygen consumptions might be due to a slower intensity progression in the present study compared to the RCT. In addition, due to practical reasons, there were two and five days of rest between sessions in the present study which also could have had a negative impact [52]. Importantly, some exercise participants in this study also expressed improvements in energy, sleep quality, and endurance that were not quantitatively assessed.

Appropriateness of supramaximal HIT was acknowledged, both by exercise participants and instructors, at least conceptually. However, the experience of suitability of the training appeared to vary depending on participants’ self-described prior experience with structured or strenuous exercise, and their motivation for engaging in the intervention. Some participants expressed confidence in their ability to assess and manage their physical limits during the sessions, drawing on previous training experiences. Instructors also reflected that individuals with such backgrounds more readily established a rhythm in the sessions and in training with the high intensity. At the same time, others, regardless of training background, appreciated the short and structured format feasible, although with a learning curve in managing the rapid and sometimes large changes in resistance at interval onset, confirmed by both the exercise instructors’ and researchers’ observations. To improve the supramaximal HIT programme, exercise participants and instructors suggested extending the training sessions or incorporating longer intervals for a higher exercise dose. Longer intervals might also improve the participants’ adherence to the programme, as previously mentioned, and simplify assessment of effort, but might also increase the perceived effort [53].

Some research indicates that the intermittent nature of interval training in adaptable and manageable bouts is an opportunity to enhance exercise self-efficacy for older adults with and without previous experience of high-intensity training [54]. Others argue that interval exercise is too complex and difficult, negatively affecting self-efficacy through perceptions of incompetence and low levels of enjoyment [20]. Individual variation in exercise-related self-efficacy and motivation, as in the current study, might be expected, since the exercise participants expressed a trade-off between a range of positive and negative sensations. Findings that largely agrees with previous findings on young inactive adults performing high-intensity training [55].

Findings from a focus group study from the beforementioned RCT of the supramaximal HIT programme align with the present study in highlighting that many older adults value the group setting as a central component of their exercise experience [56]. The group dynamics fostered social bonds and mutual accountability, which participants in both studies expressed as crucial for maintaining motivation and engagement. Furthermore, shared experiences and peer support helped mitigate challenges, such as lack of familiarity with the training format or equipment, reinforcing the motivational synergy provided by group-based supramaximal HIT [56].

A general concern for wider adoption, agreed upon by both the exercise participants and the exercise instructors, is related to health and health risks when older adults start a new form of exercise. All exercise participants in this study underwent medical screening, which they viewed as important for building confidence to push their limits. It also gave exercise instructors the reassurance needed to motivate them. Medical supervision has previously been shown to enhance the perceived safety, particularly for those unsure about whether the physical activity is suitable for them [57]. However, advocating medical screening for all older adults may also be a significant barrier for adoption of supramaximal HIT and other forms of vigorous exercise in a community setting.

A barrier to sustainability of the specific programme is the individualization of the prescribed resistance. We used a test to prescribe an absolute start intensity and provided recommendations on suitable relative intensity regarding ratings of perceived exertion. The exercise participants were then responsible for their own training intensity during the rest of the study, which is a clear shift in responsibility towards the exercise participants compared to the previous study evaluating this programme [15, 19]. This responsibility was perceived as positive and empowering. The logbooks reveal that, as a group, they did progress in absolute external intensity over time. However, it seems that the progression was a little slower and with more individual variation compared to the previous RCT, in which the absolute intensity was managed by researchers [19]. The management of a logbook, based on a test, is a further addition of complexity to the training programme. Too many parameters to keep track of during sessions and in the logbook were mentioned by the exercise instructors as limiting the programme’s sustainability. Inactive adults have also favoured moderate-intensity continuous training over interval training, in part due to it being less complicated and more familiar [55].

From a broader sustainability perspective, simplifying the programme’s complexity and addressing societal attitudes toward ageing are critical. Fridberg et al. (2025) demonstrated that participants’ engagement with supramaximal HIT involved overcoming internalised ageist stereotypes and societal expectations that undervalue older adults’ physical capabilities [56]. By successfully participating in structured group settings, participants in both the previous study evaluating the supramaximal HIT program [56], and the present one, not only reported physical benefits but also experienced a sense of empowerment and belonging. These findings suggest that empowering older adults through accessible, well-supported HIT-programmes can position them as active contributors to their health while challenging restrictive age-based norms. In the present study, the greater shift towards participant autonomy—where responsibility for intensity progression was transferred from researchers to participants—supports wider adoption by reducing logistical demands but also highlights a trade-off between fidelity and sustainability. While this autonomy can empower older adults and simplify scaling up the intervention, it underscores the need to balance flexibility with guidance to maintain consistency and outcomes. Embedding supramaximal HIT in public health strategies can normalise high-intensity exercise for older adults and foster community-level acceptance.

### Strengths and limitations

A methodological strength of our study is the use of multiple data collection methods [58], incorporating diverse perspectives from exercise participants and instructors. Another strength is the triangulation of results among researchers with and without involvement in the development of the specific programme, and with different areas of expertise (physiotherapy, cardiology, and neuroscience, as well as qualitative and quantitative analysis).

To guide our pragmatic feasibility analysis, we utilized Proctor et al.’s taxonomy for implementation outcomes [24]. Although various frameworks and taxonomies exist [59, 60], we chose this taxonomy for its comprehensive synthesis of implementation outcomes. Importantly, the taxonomy required interpretation and adaptation to fit our specific context and aim. A key strength of our approach was the predefinition of these context-specific adaptations in the coding sheet, ensuring consistency and rigour in our analysis. In our deductive analysis, we identified expressions corresponding to all implementation outcomes in the predefined coding sheet. A limitation is that we did not specify a hypothesis defining the study’s expectations on programme feasibility.

The study was conducted with a relatively homogeneous sample of exercise participants that actively volunteered to a study about exercise. Most also had some self-reported exercise experience in adult age, and they were prior to inclusion screened by a cardiologist. The training facility is in the local context large, has the equipment used to develop the programme, and it has staff with permanent positions and extensive experience. The selected participants and training facility may limit the generalizability of the findings. However, by employing the taxonomy, we aimed to increase the generalizability and transferability of the results to other settings and future implementation. Additionally, the study did not reach the pre-specified sample size [27], and therefore no formal statistical analyses were performed. Given study design and the sample size, the observed improvements should not be viewed as evidence of intervention effectiveness. However, the results indicate variation and effect in a community setting that can inform a properly powered future effectiveness study.

## Conclusions

This study demonstrates that supramaximal HIT can be implemented for older adults in a community setting, provided that adequate support is available, including individualized Watt-based absolute intensity and structured progression. This study provides valuable insights into research concerned with empowering older adults to take ownership of their intensity progression over time, enabling them to challenge their limits. However, it also highlights that the fixed structure and complexity involved in managing short intervals may hinder the programme’s broader adoption within community settings. To successfully scale up the intervention, it is crucial to simplify interval management and provide clear guidance, thereby enhancing participants’ confidence and fidelity to the programme. Moreover, it must be easy for training facilities to adopt the programme and create a sustainable framework for its long-term integration. Ultimately, the findings from this study provides valuable insight for future efforts to refine and implement supramaximal HIT programmes for older adults in community settings.

## Supplementary Information

Supplementary Material 1.

Supplementary Material 2.

## Data Availability

The datasets used and/or analysed during the current study are available from the corresponding author on reasonable request.

## Abbreviations

BREQ-2: Behavioral regulations in exercise questionnaire-2
COREQ: Consolidated criteria for reporting qualitative research
EI: Exercise instructors
EP: Exercise participants
HIT: High–intensity interval training
MAXQDA: MAX [Weber] qualitative data analysis
RPE: Rating of perceived exertion
RCT: Randomised controlled trial
S-ESES: Swedish exercise self–efficacy scale

## References

[1] Hawkins SA, Wiswell RA. Rate and mechanism of maximal oxygen consumption decline with aging. Sports Med. 2003;33:877–88. 12974656 10.2165/00007256-200333120-00002

[2] Holmlund T, Ekblom B, Börjesson M, Andersson G, Wallin P, Ekblom-Bak E. Association between change in cardiorespiratory fitness and incident hypertension in Swedish adults. Eur J Prev Cardiolog. 2021;13:1515–22. 10.1177/2047487320942997 32812803

[3] Suetta C, Haddock B, Alcazar J, Noerst T, Hansen OM, Ludvig H, et al. The Copenhagen sarcopenia study: lean mass, strength, power, and physical function in a Danish cohort aged 20–93 years. J Cachexia Sarcopenia Muscle. 2019;10:1316–29. 31419087 10.1002/jcsm.12477 PMC6903448

[4] Whelton SP, McAuley PA, Dardari Z, Orimoloye OA, Michos ED, Brawner CA, et al. Fitness and mortality among persons 70 years and older across the spectrum of cardiovascular disease risk factor burden: the FIT project. Mayo Clin Proc. 2021;96:2376–85. 34366139 10.1016/j.mayocp.2020.12.039

[5] Bull FC, Al-Ansari SS, Biddle S, Borodulin K, Buman MP, Cardon G, et al. World health organization 2020 guidelines on physical activity and sedentary behaviour. Br J Sports Med. 2020;54:1451–62. 33239350 10.1136/bjsports-2020-102955 PMC7719906

[6] Townsend N, Wickramasinghe K, Williams J, Bhatnagar P, Rayner M. Levels of physical activity. In Physical Activity Statistics 2015. Edited by Dicks E London: British Heart Foundation; 2015:12–37.

[7] Franco MR, Tong A, Howard K, Sherrington C, Ferreira PH, Pinto RZ, et al. Older people’s perspectives on participation in physical activity: a systematic review and thematic synthesis of qualitative literature. Br J Sports Med. 2015;49:1268–76. 25586911 10.1136/bjsports-2014-094015

[8] Gibala MJ, Little JP, MacDonald MJ, Hawley JA. Physiological adaptations to low-volume, high-intensity interval training in health and disease. J Physiol. 2012;590:1077–84. 22289907 10.1113/jphysiol.2011.224725 PMC3381816

[9] Campbell WW, Kraus WE, Powell KE, Haskell WL, Janz KF, Jakicic JM, et al. High-Intensity interval training for cardiometabolic disease prevention. Med Sci Sports Exerc. 2019;51:1220. 31095079 10.1249/MSS.0000000000001934 PMC6777577

[10] Wu Z-J, Wang Z-Y, Gao H-E, Zhou X-F, Li F-H. Impact of high-intensity interval training on cardiorespiratory fitness, body composition, physical fitness, and metabolic parameters in older adults: A meta-analysis of randomized controlled trials. Exp Gerontol. 2021;150:111345. 33836261 10.1016/j.exger.2021.111345

[11] Bouaziz W, Malgoyre A, Schmitt E, Lang P-O, Vogel T, Kanagaratnam L. Effect of high-intensity interval training and continuous endurance training on peak oxygen uptake among seniors aged 65 or older: A meta-analysis of randomized controlled trials. Int J Clin Pract. 2020;74:e13490. 32083390 10.1111/ijcp.13490

[12] Bartlett JD, Close GL, MacLaren DPM, Gregson W, Drust B, Morton JP. High-intensity interval running is perceived to be more enjoyable than moderate-intensity continuous exercise: implications for exercise adherence. J Sports Sci. 2011;29:547–53. 21360405 10.1080/02640414.2010.545427

[13] Coates AM, Joyner MJ, Little JP, Jones AM, Gibala MJ. A perspective on High-Intensity interval training for performance and health. Sports Med. 2023;53:85–96. 37804419 10.1007/s40279-023-01938-6 PMC10721680

[14] Hedlund M, Lindelöf N, Johansson B, Boraxbekk C-J, Rosendahl E. Development and feasibility of a regulated, supramaximal High-Intensity training program adapted for older individuals. Front Physiol. 2019;10:590. 10.3389/fphys.2019.00590 PMC6536694 31164835

[15] Simonsson E, Levik Sandström S, Hedlund M, Holmberg H, Johansson B, Lindelöf N, et al. Effects of controlled supramaximal High-Intensity interval training on cardiorespiratory fitness and global cognitive function in older adults: the Umeå HIT Study—A randomized controlled trial. Lipsitz LA, editor. J Gerontol Biol Sci Med Sci. 2023;78:1581–90. 10.1093/gerona/glad070 PMC10460559 36972981

[16] Adamson S, Lorimer R, Cobley J, Lloyd R, Babraj J. High intensity training improves health and physical function in middle aged adults. Biology (Basel). 2014;3:333–44. 24833513 10.3390/biology3020333 PMC4085611

[17] Adamson S, Kavaliauskas M, Yamagishi T, Phillips S, Lorimer R, Babraj J. Extremely short duration sprint interval training improves vascular health in older adults. Sport Sci Health. 2019;15:123–31.

[18] Allen JR, Satiroglu R, Fico B, Tanaka H, Vardarli E, Luci JJ, et al. Inertial load power cycling training increases muscle mass and aerobic power in older adults. Med Sci Sports Exerc. 2021;53:1188–93. 33433149 10.1249/MSS.0000000000002588

[19] Frykholm E, Simonsson E, Levik Sandström S, Hedlund M, Holmberg H, Johansson B, et al. Applicability of a supramaximal high-intensity interval training program for older adults previously not engaged in regular exercise; analyses of secondary outcomes from the Umeå HIT study. Psychol Sport Exerc. 2024;73:102647. 38604572 10.1016/j.psychsport.2024.102647

[20] Hardcastle SJ, Ray H, Beale L, Hagger MS. Why sprint interval training is inappropriate for a largely sedentary population. Front Psychol. 2014;5:01505. 10.3389/fpsyg.2014.01505 PMC4274872 25566166

[21] Biddle SJH, Batterham AM. High-intensity interval exercise training for public health: a big HIT or shall we HIT it on the head? Int J Behav Nutr Phys Activity. 2015;12:95. 10.1186/s12966-015-0254-9 PMC4506613 26187579

[22] Ford I, Norrie J. Pragmatic trials. N Engl J Med. 2016;375:454–63. 27518663 10.1056/NEJMra1510059

[23] Skivington K, Matthews L, Simpson SA, Craig P, Baird J, Blazeby JM, et al. A new framework for developing and evaluating complex interventions: update of medical research Council guidance. BMJ. 2021;374:n2061. 34593508 10.1136/bmj.n2061 PMC8482308

[24] Proctor E, Silmere H, Raghavan R, Hovmand P, Aarons G, Bunger A, et al. Outcomes for implementation research: conceptual distinctions, measurement challenges, and research agenda. Adm Policy Ment Health. 2011;38:65–76. 20957426 10.1007/s10488-010-0319-7 PMC3068522

[25] Fetters MD, Curry LA, Creswell JW. Achieving integration in mixed methods Designs—Principles and practices. Health Serv Res. 2013;48:2134–56. 24279835 10.1111/1475-6773.12117 PMC4097839

[26] Morse JM, Cheek J. Making room for Qualitatively-Driven Mixed-Method research. Qual Health Res. 2014;24:3–5. 24448383 10.1177/1049732313513656

[27] Frykholm E, Pettersson B, Frankel J, Sandstrom S, Hedlund M, Johansson B et al. (2023) High-Intensity training for older adults– from the laboratory to the gym. Available from: https://osf.io/b7t2g. Accessed 13 June 2024.

[28] Tong A, Sainsbury P, Craig J. Consolidated criteria for reporting qualitative research (COREQ): a 32-item checklist for interviews and focus groups. Int J Qual Health Care. 2007;19:349–57. 17872937 10.1093/intqhc/mzm042

[29] Borg G. Ratings of perceived exertion and heart rates during Short-Term cycle exercise and their use in a new cycling strength test**. Int J Sports Med. 1982;03:153–8. 10.1055/s-2008-1026080 7129724

[30] Borg G. Borg’s Perceived exertion and pain scales. Champaign, Ill: Human Kinetics; 1998.

[31] Björkman F, Ekblom-Bak E, Ekblom Ö, Ekblom B. Validity of the revised Ekblom bak cycle ergometer test in adults. Eur J Appl Physiol. 2016;116:1627–38. 27311582 10.1007/s00421-016-3412-0 PMC4983286

[32] Olsson SJG, Ekblom Ö, Andersson E, Börjesson M, Kallings LV. Categorical answer modes provide superior validity to open answers when asking for level of physical activity: A cross-sectional study. Scand J Public Health. 2016;44:70–6. 26392418 10.1177/1403494815602830

[33] Ahlström I, Hellström K, Emtner M, Anens E. Reliability of the Swedish version of the exercise Self-Efficacy scale (S-ESES): a test–retest study in adults with neurological disease. Physiother Theory Pract. 2015;31:194–9. 25418018 10.3109/09593985.2014.982776

[34] Kroll T, Kehn M, Ho P-S, Groah S. The SCI exercise Self-Efficacy scale (ESES): development and psychometric properties. Int J Behav Nutr Phys Act. 2007;4:34. 17760999 10.1186/1479-5868-4-34 PMC2034591

[35] Lindwall M, Ivarsson A, Weman-Josefsson K, Jonsson L, Ntoumanis N, Patrick H, et al. Stirring the motivational soup: within-person latent profiles of motivation in exercise. Int J Behav Nutr Phys Act. 2017;14:4. 28088208 10.1186/s12966-017-0464-4 PMC5237570

[36] Markland D, Tobin V. A modification to the behavioural regulation in exercise questionnaire to include an assessment of amotivation. J Sport Exerc Psychol. 2004;26:191–6.

[37] Weman-Josefsson K, Lindwall M, Ivarsson A. Need satisfaction, motivational regulations and exercise: moderation and mediation effects. Int J Behav Nutr Phys Act. 2015;12:67. 25990492 10.1186/s12966-015-0226-0 PMC4489042

[38] Wilson PM, Sabiston CM, Mack DE, Blanchard CM. On the nature and function of scoring protocols used in exercise motivation research: an empirical study of the behavioral regulation in exercise questionnaire. Psychol Sport Exerc. 2012;13:614–22.

[39] Teixeira PJ, Carraça EV, Markland D, Silva MN, Ryan RM. Exercise, physical activity, and self-determination theory: A systematic review. Int J Behav Nutr Phys Act. 2012;9:78. 22726453 10.1186/1479-5868-9-78 PMC3441783

[40] Strandell T. Circulatory studies on healthy old men with special reference to the limitation of the maximal physical working capacity. Acta Med Scand Suppl. 1964;414:SUPPL. 14161322

[41] Graneheim UH, Lindgren B-M, Lundman B. Methodological challenges in qualitative content analysis: A discussion paper. Nurse Education Today. 2017;56:29–34. 28651100 10.1016/j.nedt.2017.06.002

[42] Elo S, Kyngäs H. The qualitative content analysis process. J Adv Nurs. 2008;62:107–15. 18352969 10.1111/j.1365-2648.2007.04569.x

[43] Lindgren B-M, Lundman B, Graneheim UH. Abstraction and interpretation during the qualitative content analysis process. Int J Nurs Stud. 2020;108:103632. 32505813 10.1016/j.ijnurstu.2020.103632

[44] R Core Team. R: A language and environment for statistical computing [Internet], Vienna. Austria: R Foundation for Statistical Computing; 2023. Available from: https://www.R-project.org/. Accessed 27 Nov 2023.

[45] Posit team. RStudio: Integrated development environment for R [Internet]. Posit Software, PBC. 2022. Available from: http://www.posit.co/. Accessed 22 Feb 2024.

[46] Wickham H, Averick M, Bryan J, Chang W, McGowan L, François R, et al. Welcome to the tidyverse. J Open Source Softw. 2019;4:1686.

[47] Marriott CFS, Petrella AFM, Marriott ECS, Boa Sorte Silva NC, Petrella RJ. High-Intensity interval training in older adults: a scoping review. Sports Med - Open. 2021;7:49. 34279765 10.1186/s40798-021-00344-4 PMC8289951

[48] Burn NL, Weston M, Atkinson G, Graham M, Weston KL. Brief exercise at work (BE@Work): A Mixed-Methods pilot trial of a workplace High-Intensity interval training intervention. Front Sports Act Living. 2021;3:699608. 10.3389/fspor.2021.699608 PMC8282817 34278300

[49] Lunt H, Draper N, Marshall HC, Logan FJ, Hamlin MJ, Shearman JP, et al. High intensity interval training in a real world setting: A randomized controlled feasibility study in overweight inactive adults, measuring change in maximal oxygen uptake. PLoS ONE. 2014;9:e83256. 24454698 10.1371/journal.pone.0083256 PMC3890270

[50] Kinnafick F-E, Thøgersen-Ntoumani C, Shepherd SO, Wilson OJ, Wagenmakers AJM, Shaw CS. In it together: A qualitative evaluation of participant experiences of a 10-Week, Group-Based, workplace HIIT program for insufficiently active adults. J Sport Exerc Psychol. 2018;40:10–9. 29521569 10.1123/jsep.2017-0306

[51] Frykholm E, Hedlund M, Becker C, Holmberg H, Johansson B, Klenk J, et al. Effects of controlled supramaximal high-intensity interval training on muscle capacities and physical functions for older adults: analysis of secondary outcomes from the Umeå HIT study—a randomised controlled trial. Age Ageing. 2024;53:afae226. 39396911 10.1093/ageing/afae226 PMC11471315

[52] Herbert P, Grace FM, Sculthorpe NF. Exercising caution: prolonged recovery from a single session of High-Intensity interval training in older men. J Am Geriatr Soc. 2015;63:817–8. 25900496 10.1111/jgs.13365

[53] Metcalfe RS, Williams S, Fernandes GS, Astorino TA, Stork MJ, Phillips SM, et al. Affecting effects on affect: the impact of protocol permutations on affective responses to sprint interval exercise; A systematic review and Meta-Analysis of pooled individual participant data. Front Sports Act Living. 2022;4:815555. 35252858 10.3389/fspor.2022.815555 PMC8891702

[54] Jung ME, Bourne JE, Little JP. Where does HIT fit?? An examination of the affective response to High-Intensity intervals in comparison to continuous Moderate- and continuous Vigorous-Intensity exercise in the exercise Intensity-Affect continuum. PLoS ONE. 2014;9:e114541. 25486273 10.1371/journal.pone.0114541 PMC4259348

[55] Stork MJ, Williams TL, Martin Ginis KA. Unpacking the debate: A qualitative investigation of first-time experiences with interval exercise. Psychol Sport Exerc. 2020;51:101788.

[56] Fridberg H, Wiklund M, Snellman F, Rosendahl E, Hedlund M, Boraxbekk C-J, et al. Negotiating a physically active life in tune with ageing: a grounded theory study of older persons’ experiences of participating in high-intensity interval training. BMC Geriatr. 2025;25:11. 39755610 10.1186/s12877-024-05635-5 PMC11699672

[57] Devereux-Fitzgerald A, Powell R, Dewhurst A, French DP. The acceptability of physical activity interventions to older adults: A systematic review and meta-synthesis. Soc Sci Med. 2016;158:14–23. 27104307 10.1016/j.socscimed.2016.04.006

[58] Shenton AK. Strategies for ensuring trustworthiness in qualitative research projects. EFI. 2004;22:63–75.

[59] Glasgow RE, Vogt TM, Boles SM. Evaluating the public health impact of health promotion interventions: the RE-AIM framework. Am J Public Health. 1999;89:1322–7. 10474547 10.2105/ajph.89.9.1322 PMC1508772

[60] Rogers EM. Diffusion of innovations, 5. ed. New York: Free press; 2003.

