# Supplementary material for: Supramaximal high-intensity interval training for older adults in a community setting: a pragmatic feasibility study
Source: Eur Rev Aging Phys Act. 2025 Jul 28;22:13. doi: 10.1186/s11556-025-00379-6 (PMC12302583; doi:10.1186/s11556-025-00379-6)
Supplement: Supplementary file 1 — Supplementary Material 1. [file 11556_2025_379_MOESM1_ESM.docx]

## Interview Guide for Exercise Participants

**Background**

- What made you decide to join this study?
- Could you tell me a little about your previous exercise habits?
- Have you previously participated in group training or cycling classes?
  - If yes: Do you feel there are any advantages or disadvantages of a HIT training session compared to a regular cycling class?
- What expectations did you have when joining this study?
  - Do you feel these expectations have been met?
- How has your motivation for training been over the course of the study?
- Do you feel that this type of exercise suits you?
  - Why or why not?
- What do you think your family or friends think about your training?
  - Have they been involved or engaged in your training in any way?

**Adherence**

- To what extent have you been able to participate in the training sessions during this period?

**Training Programme**

- How did you find training with these supramaximal cycling intervals?
- How did you experience the responsibility of finding the right resistance on the bike?
  - Has anything changed in how you experience the sessions over the course of the 25 sessions?
- Have you encountered any challenges that made it difficult to complete the training?
  - If so, what did you do when they occurred?
- Is there anything you found helpful in making it easier to complete the training?
- To what extent did you feel that the programme and instructors sufficiently supported you to successfully manage and carry out the training sessions on your own?
- Is there anything else you think could have made it easier to complete the training?
- Have you thought of any particular advantages or disadvantages of this type of training?
- Who do you think this type of training might be especially suitable or unsuitable for?

**Health**

- Has participating in the study affected your health in any way?
  - Positive/negative effects?
- Has participation in the study affected how you perform daily activities, such as shopping or dressing yourself?

**Sustainability**

- If this type of training were available at a fitness centre, would you want to continue with it?
- Have you started any other type of exercise during these 12 weeks?
- Do you plan to start any other type of exercise after this training period?

## Interview Guide for Exercise Instructors

**Acceptance**

• How have you experienced leading supramaximal HIT cycling classes?

• What similarities and differences do you notice between leading a HIT cycling class and a regular cycling class?

• To what extent did you feel you had the ability to influence the format of the sessions?

- Has this changed over the 12-week period?

**Adherence**

• How well do you think the participants were able to perform the exercises as intended?

• How did the participants seem to respond to the training format/protocol?

• Have you observed any changes in participants’ motivation and engagement over the training period?

**Recipients**

• Is there anything you have learned from working with a supramaximal HIT protocol and leading this type of group exercise class?

• How was it to lead a class exclusively for individuals over 65?

• If this type of training were to continue at the facility, could you share your thoughts on continuing to lead this type of class?

**Implementation**

• Do you feel you received adequate support during the rollout of the group training?

• Do you feel that you were prepared/had the information you needed to lead supramaximal HIT group training?

**Execution**

• Did you need to assist participants in understanding how to perform the exercises?

• Did you find it necessary to modify the execution or instructions of the exercises at any point during the intervention?

- If so, why?

• Have you had to make any individual adjustments for certain participants?

• Have you encountered any particular challenges throughout the training period?

- If so, how did you address them?

• What advantages or disadvantages do you see with this type of training? Feel free to elaborate.

• Has anything surprised you in this process?

• The sessions are fairly structured and brief in duration. Have you experimented with different ways to add variety or instruct the classes?

• Do you have any thoughts on how the training protocol could be improved?

**External Context**

• How do you view supramaximal HIT training as a future class that the facility could provide for seniors?

• To what extent do you feel that this type of training complements the regular offerings at the training facility?

• Have the facilities/equipment worked well for the intervention?
