# Supplementary material for: Supramaximal high-intensity interval training for older adults in a community setting: a pragmatic feasibility study
Source: Eur Rev Aging Phys Act. 2025 Jul 28;22:13. doi: 10.1186/s11556-025-00379-6 (PMC12302583; doi:10.1186/s11556-025-00379-6)
Supplement: Supplementary file 2 — Supplementary Material 2. [file 11556_2025_379_MOESM2_ESM.docx]

Coding sheet

Coding sheet based on the taxonomy of implementation outcomes by Proctor et al.
(DOI: 10.1007/s10488-010-0319-7) adapted for the analysis of the implementation of supramaximal HIT in a community-based training facility.

| Construct | Definition | Adapted definition |
| --- | --- | --- |
| Adoption | Adoption is defined as the intention, initial decision, or action to try or employ an innovation or evidence-based practice. Adoption may also be referred to as ‘uptake’. | Participants’ intentions, initial decisions, or actions to try or employ supramaximal HIT.  **Inclusion criteria:** Includes statements about factors influencing older individuals’ decisions to initiate HIT training, including their initial motivations and intentions to explore HIT as a training method.  **Exclusion criteria:** Excludes statements regarding general attitudes of training for older adults, or statements about the execution or implementation of supramaximal HIT. |
| Appropriateness | Appropriateness is the perceived fit, relevance, or compatibility of the innovation or evidence-based practice for a given practice setting, provider, or consumer, and/or perceived fit of the innovation to address a particular issue or problem.   ‘Appropriateness’ is conceptually similar to ‘acceptability’. We preserve a distinction because a given treatment may be perceived as appropriate but not acceptable, and vice versa. | Instructors’ perceptions of the perceived fit, relevance, or compatibility of supramaximal HIT in a gym setting; how well HIT is perceived as a suitable form of physical activity for older adults.  **Inclusion criteria:** Includes statements of how supramaximal HIT fits within the existing gym organization, and can be adapted/tailored to the gym’s needs. Also includes the individual’s perceived fit of supramaximal HIT as a suitable form of physical activity for themselves or older adults, including preferences, personal circumstances, values, norms, risks and needs.  **Exclusion criteria:** Excludes statements of supramaximal HIT as easy or difficult to use or understand. Code to feasibility. |
| Acceptability | Acceptability is the perception among stakeholders that a specific treatment, service, or innovation is satisfactory or agreeable. Unlike general service satisfaction, which covers broader service aspects (e.g., waiting times or environment), acceptability focuses specifically on the treatment itself, considering factors such as content and ease of use. It can be evaluated from different stakeholders’ perspectives, such as administrators and consumers, and may change as they gain experience with the treatment. | Instructors’ and participants’ perceptions of supramaximal HIT as an agreeable, satisfactory, and motivating form of exercise.  **Inclusion criteria:** Includes statements related to attitudes towards supramaximal HIT, and experiences of the content and complexity of instructing or carrying out supramaximal HIT.  **Exclusion criteria:** Excludes statements related to the implementation of supramaximal HIT withing the organization or gym setting. |
| Feasibility | Feasibility is defined as the extent to which a new treatment, or an innovation, can be successfully used or carried out within a given agency or setting.   While feasibility is related to appropriateness, the two constructs are conceptually distinct. For example, a programme may be appropriate for a service setting in that it is compatible with the setting’s mission or service mandate, but may not be feasible due to resource or training requirements. | The instructors’ experiences of carrying out supramaximal HIT, and the participants’ experiences of participating in supramaximal HIT.  **Inclusion criteria:** Includes statements of actions and routines for implementing supramaximal HIT and experiences related to instructing or carrying out the training.  **Exclusion criteria:** Excludes statements regarding the ease or difficulty of working in line with the instructions/concept of supramaximal HIT. Code to fidelity. |
| Fidelity | Fidelity is defined as the degree to which an intervention was implemented as it was prescribed in the original protocol or as it was intended by the programme developer. Can include dimensions of adherence to the programme protocol, dose or amount of programme delivered, and quality of programme delivery. | The instructors’ perceptions of how the supramaximal HIT was carried out as intended, and the participants’ experiences of carrying out supramaximal HIT according to instruction.  **Inclusion criteria:** Includes statements of the perceived difficulty of instructing or carrying out supramaximal HIT according to the protocol.  **Exclusion criteria:** Excludes statements of routines of carrying out the training or general perceptions. |
| Sustainability | Sustainability is defined as the extent to which a newly implemented treatment is maintained or institutionalized within a service setting’s ongoing, stable operations.   The proposed definition incorporates aspects of integration of a given programme within an organization’s culture through policies and practices, and distinguishes three stages that determine institutionalization: (1) passage (a single event such as transition from temporary to permanent funding), (2) cycle or routine (i.e., repetitive reinforcement of the importance of the evidence-based intervention by including it in organizational or community procedures and behaviours, such as the annual budget and evaluation criteria), and (3) niche saturation (the extent to which an evidence-based intervention is integrated into all subsystems of an organization). | The older adults’ and instructors’ perceptions of how well HIT training can be transitioned or institutionalized within the training facility’s ongoing, stable operations.  **Inclusion criteria:** Includes statements regarding passage, i.e., how the supramaximal HIT can be or is intended to be integrated in the gym organization’s practice, or the participants’ intentions or wishes to continue with supramaximal HIT.  **Exclusion criteria:** Excludes statements regarding changes in perception of appropriateness or acceptability. Code respectively. |
| Cost | Cost (incremental or implementation cost) is defined as the cost impact of an implementation effort. It varies based on the treatment’s complexity, the implementation strategy used, and the service setting, such as a small clinic versus a large hospital. | NA for the study. |
| Penetration | Penetration is defined as the integration of a practice within a service setting and its subsystems. Penetration also can be calculated in terms of the number of providers who deliver a given service or treatment, divided by the total number of providers trained in or expected to deliver the service. From a service system perspective, the construct is also similar to ‘reach’ in the RE-AIM framework. | NA for the study. |
